# Impact of expressive intentions on upper-body kinematics in two expert pianists

**DOI:** 10.3389/fpsyg.2024.1504456

**Published:** 2025-01-13

**Authors:** Craig Turner, Robin Mailly, Fabien Dal Maso, Felipe Verdugo

**Affiliations:** Laboratoire de Simulation et Modélisation du Mouvement, École de Kinésiologie et des Sciences de l'Activité Physique, Université de Montréal, Montréal, QC, Canada

**Keywords:** piano performance biomechanics, music expression, musculoskeletal injury, movement smoothness, posture, range of motion, inertial measurement units, embodied cognition

## Abstract

**Introduction:**

Expression is a key aspect of music performance. Studies on pianists’ gestures and expression have mainly documented the impact of their expressive intentions on proximal segments and head linear kinematics. It remains unclear how pianists’ expressive intentions influence joint angular kinematics as well as exposure to risk factors of injury, such as poor overall posture and distal jerky movements, two kinematic factors linked to injury. The first objective of this exploratory case study was to analyze the influence of pianists’ expressive intentions on proximal and distal joint range of motion (ROM) across different musical contexts. The second objective was to evaluate the impact of expressive intentions on the posture of joints that are commonly injured in pianists, as well as distal joint angular jerk. Methods: Two expert pianists (P1 and P2) performed six musical excerpts (E1–E6) in two experimental conditions: normal condition (including expressive intentions) and the control condition (strictly playing the composer’s notations written in the score with no subjective interpretation). An inertial measurement unit system recorded upper body kinematics.

**Methods:**

Two expert pianists (P1 and P2) performed six musical excerpts (E1–E6) in two experimental conditions: normal condition (including expressive intentions) and the control condition (strictly playing the composer’s notations written in the score with no subjective interpretation). An inertial measurement unit system recorded upper body kinematics.

**Results and discussion:**

Both proximal and distal joint ROM increased when pianists incorporated expressive intentions. Participants exhibited more static, non-neutral wrist postures when incorporating expressive intentions (right and left wrist for P1 and P2, respectively), suggesting an increased risk of distal injury. On the contrary, the thorax exhibited more dynamic, neutral flexion postures, suggesting a reduced risk of proximal injury. These results suggest that expressive intentions may impact proximal and distal postures differently. Incorporating expressive intentions also led to jerkier, less smooth wrist movements in lyrical, non-virtuosic musical excerpts (E1–E4). However, in more virtuosic excerpts (E5–E6), there were generally no differences between conditions. Spatiotemporal constraints might explain these discrepancies between non-virtuosic and virtuosic musical excerpts. These results provide evidence of the impact of expressive intentions on the entire kinematic chain, while highlighting the implications of the subjective dimension of music expression in relation to exposure to risk factors of injury.

## 1 Introduction

In music performance, expressive intentions are a performer’s individualized deliberate decisions for the purpose of conveying specific artistic content from a piece of music (Leman and Maes, 2014). Based on the embodied cognition theory, pianists’ expressive intentions influence how they move and interact with the piano, which in turn further shapes the expressive intentions themselves (Leman et al., 2018). Therefore, gesture is a medium through which pianists can not only create sound but also convey and shape expressive intentions. Studies focusing on the impact of expressive intentions on musicians’ gestures have required participants to perform excerpts from musical repertoire using different expressive conditions such as “normal,” “exaggerated,” and “deadpan” for the piano (Massie-Laberge et al., 2018, 2019; Thompson and Luck, 2012), clarinet (Wanderley et al., 2005), violin (Davidson, 1993), and marimba (Broughton and Stevens, 2009). These studies have in general categorized different types of gestures as sound-producing gestures, sound-facilitating gestures, communicative gestures, and sound-accompanying gestures (Dahl et al., 2010; Jensenius et al., 2010). In the case of piano performance, sound-producing and sound-facilitating gestures are better categorized as distinct gestural functions rather than distinct gestures, as these two functions can be embedded in a single gesture that incorporates the entire kinematic chain (Mailly et al., 2024a).

One primary metric for assessing gestural differences between expressive conditions in the relevant literature is the “quantity of motion” of markers placed on participants’ skin, which has been defined as the cumulative distance a marker has moved over a certain time. For instance, it has been documented that markers placed on the head (Castellano et al., 2008; Massie-Laberge et al., 2019; Thompson and Luck, 2012), trunk, and shoulder girdle (Massie-Laberge et al., 2019; Thompson and Luck, 2012) travel greater distances when playing in expressive conditions. Markers placed on distal segments, such as the hands, are reported to be less affected in expressive conditions due to their role in sound production (Thompson and Luck, 2012). Quantifying how much a marker placed on the body moves, however, does not necessarily determine which parts of the kinematic chain are responsible for the movement. For example, in piano performance, hand key-attack and release movements could be generated from the wrist, elbow, shoulder, or trunk (Verdugo et al., 2020). Additionally, changes in trunk joint angles could equally be responsible for greater head linear motion rather than just changes in neck joint motion. Expressive intentions have been shown to also influence proximal and distal muscle activity (Mailly et al., 2024b). It is therefore unclear which joints of the kinematic chain in pianists are influenced by expressive intentions as only marker linear (also referred to as translation) kinematics have been investigated. Only three studies that addressed the impact of expressive intentions on pianists’ kinematics have considered joint angular kinematics; two studies analyzed neck, spine, and trunk angles (Wong et al., 2022, 2024) while the other studied angular excursions (i.e., range of motion) of the upper arm and trunk (Nakahara et al., 2010). No research has addressed the entire upper-body kinematic chain. Analyzing joint angular kinematics could determine how movements of proximal and distal joints are influenced by expressive intentions. Specifically, joint range of motion (ROM) can indicate whether pianists’ joints move more or less throughout musical excerpts. This could further our understanding of the embodiment process of music expression-related parameters in piano performance.

Investigating the relationship between expressive intentions and pianists’ joint kinematics in a variety of musical contexts may also provide insights on mechanisms of performance-related musculoskeletal disorders (PRMDs). Amongst pianists, point prevalence of PRMDs is 72% (Allsop and Ackland, 2010). Poor posture and jerky movements are two kinematic risk factors of PRMDs. In terms of poor posture, playing in non-neutral or static postures is a reported risk factor of injury in musicians (Garnett et al., 2022). Furthermore, static non-neutral postures may also increase exposure to risk of PRMDs in the most frequently injured areas of pianists, such as the wrists, shoulders, neck, and upper back (Bruno et al., 2008; Furuya et al., 2006). Dynamic postures, where the body is moving, are considered beneficial for reducing exposure to risks of injuries by distributing mechanical loading across joints. This is similar to theories surrounding movement variability, which is thought to distribute mechanical loading across joints and reduce exposure to risk of injury, especially during repetitive tasks (Hamill et al., 2012). Only one study suggested that playing in a “deadpan” condition (i.e., participants perform with as few expressive features as possible, such as minimal variations in tempo or sound intensity) led to pianists having a more upright, neutral back posture (Wong et al., 2022). In terms of jerk, the time derivative of acceleration, jerky movements increase the risk of injury since they involve greater changes in force on the joint (i.e., greater joint torques) and thus more mechanical loading. On the contrary, smooth movements have been associated with reducing exposure to risk of injury. Jerk is a common metric of movement smoothness, where a smaller jerk indicates greater movement smoothness. Distal smoothness could be an interesting avenue to explore in relation to risk of injury not only because injuries are most common in the wrists and forearms (Bruno et al., 2008) but also because distal segments such as the hand and forearm might be more affected by keystroke contact forces in piano performance. Smaller/distal segments might also be more affected by jerky movements compared to proximal/ larger segments because piano performance is a low intensity but highly repetitive activity. In addition, jerk-related metrics have been identified as important potential predictors of pianists’ muscle fatigue (Goubault et al., 2023), an important risk factor of PRMDs. Movement smoothness and jerk have also been used as a measure of movement efficiency and control in research on people with neurological disorders during rehabilitation (Gutiérrez et al., 2020), as a measure of muscle fatigue (Goubault et al., 2023; Mohr and Federolf, 2022; Zhang et al., 2019), and as a measure of performance in athletes (Choi et al., 2014; Ganzevles et al., 2023) and dancers (Bronner and Shippen, 2015). Additionally, smoothness has been used to address movement fluency in cellists and drummers (Gonzalez-Sanchez et al., 2019). To our knowledge, one study used smoothness to measure the synchronicity of head movements during piano duets (Bishop and Goebl, 2018) while a small section of a thesis assessed finger joint smoothness (Pilkov, 2024). As a result, further investigations on the influence of expressive intentions on both posture and movement smoothness could help increase knowledge on the exposure to risk factors of pianists’ PRMDs.

The main goal of this exploratory case study was to analyze pianists’ joint angle kinematics in relation to expressive intentions in a variety of musical contexts, including virtuosic (higher technical skills/greater difficulty) and less-virtuosic types of excerpts. The specific objectives were: (1) to analyze the influence of pianists’ expressive intentions on proximal and distal joint ROM across different musical contexts and; (2) to evaluate the impact of expressive intentions on two kinematic risk factors of PRMDs: non-neutral static posture of joints that are commonly injured in pianists (wrists, scapulae, neck, and back) and distal joint angular jerk. We hypothesized that the kinematic variables measured would be affected by expressive intentions not only at proximal but also at distal joints due to the interdependent nature of multi-joint movements, as shown in studies on pianists’ multi-joint gestures (Turner et al., 2022, 2023; Verdugo et al., 2020; Verdugo et al., 2022a). Given the scope of the current study, which focuses on the impact of expressive intentions on pianists’ joint kinematics (ROM, posture, and jerk), expressive intentions were not analyzed in terms of musical outcomes (i.e., musical parameters controlled by the performers).

## 2 Methods

### 2.1 Participants

Two expert pianists (1 female and 1 male; both right hand dominant) were recruited for this case study. Participant one (P1; female) held a doctoral degree in piano performance and was a prize winner of national and international music competitions while participant 2 (P2; male) was pursuing a doctoral degree in piano performance. The research protocol was approved by the Université de Montréal ethics committee (No. 2021–1,380). After being informed of the protocol and data collection procedures, participants gave written informed consent.

### 2.2 Experimental protocol

Participants performed six musical excerpts (E1–E6) from classical piano repertoire (all excerpts are shown in Supplementary material). E1–E4 were categorized as “lyrical excerpts” that were less technically demanding compared to E5–E6, the more “virtuosic excerpts.” The excerpts were:

- E1. Ballade Op. 23 No. 1 in G minor [measures: 1–9] by F. Chopin;
- E2. Intermezzo Op. 118 No. 2 in A major [measures: 17–34] by J. Brahms;
- E3. Polonaise-Fantasy Op. 61 in A-flat major [measures: 181–199] by F. Chopin;
- E4. Sonata Op. 111 in C minor, 1st movement [measures: 1–16] by L. v. Beethoven;
- E5. Sonata Op. 111 in C Minor, 1st movement [measures: 21–29] by L. v. Beethoven;
- E6. Concerto for Piano Op. 15 No.1 in D minor, 3rd movement [measures: 1–8] by J. Brahms

Two months prior to data collection, the musical scores were sent to the participants and individual interviews were conducted with participants to define the two experimental conditions: with expressive intentions (hereafter normal condition) and without expressive intentions (hereafter control condition). Based on the interview analysis (which is not addressed in the present article), in the control condition participants were instructed to perform the excerpt by focusing only on the musical features written by the composer, avoiding personal or subjective interpretations of the text, and playing the music as objectively as possible. Specifically, they were instructed to avoid any manipulation of musical features that were not literally written by the composer, and therefore, were not related to their personal interpretation of the score. In the normal condition, participants were asked to perform the excerpts as if in a concert or standard performance setting. The sustain pedal, which is a central element of piano tone control, is not usually specified by the composer, and its use varies according to different pianos, room acoustics, and pianists’ preferences. Therefore, in both conditions, pianists could use the sustain pedal as desired. After data collection equipment was placed on participants and calibrated, the pianists were given 10 min to warm-up. Bench height and position were adjusted based on personal preference. The order of excerpts and conditions were randomized, with each condition performed twice, labelled as trials A and B. Participants could repeat each trial until they were satisfied with their performance.

### 2.3 Instrumentation

**Figure 1:**
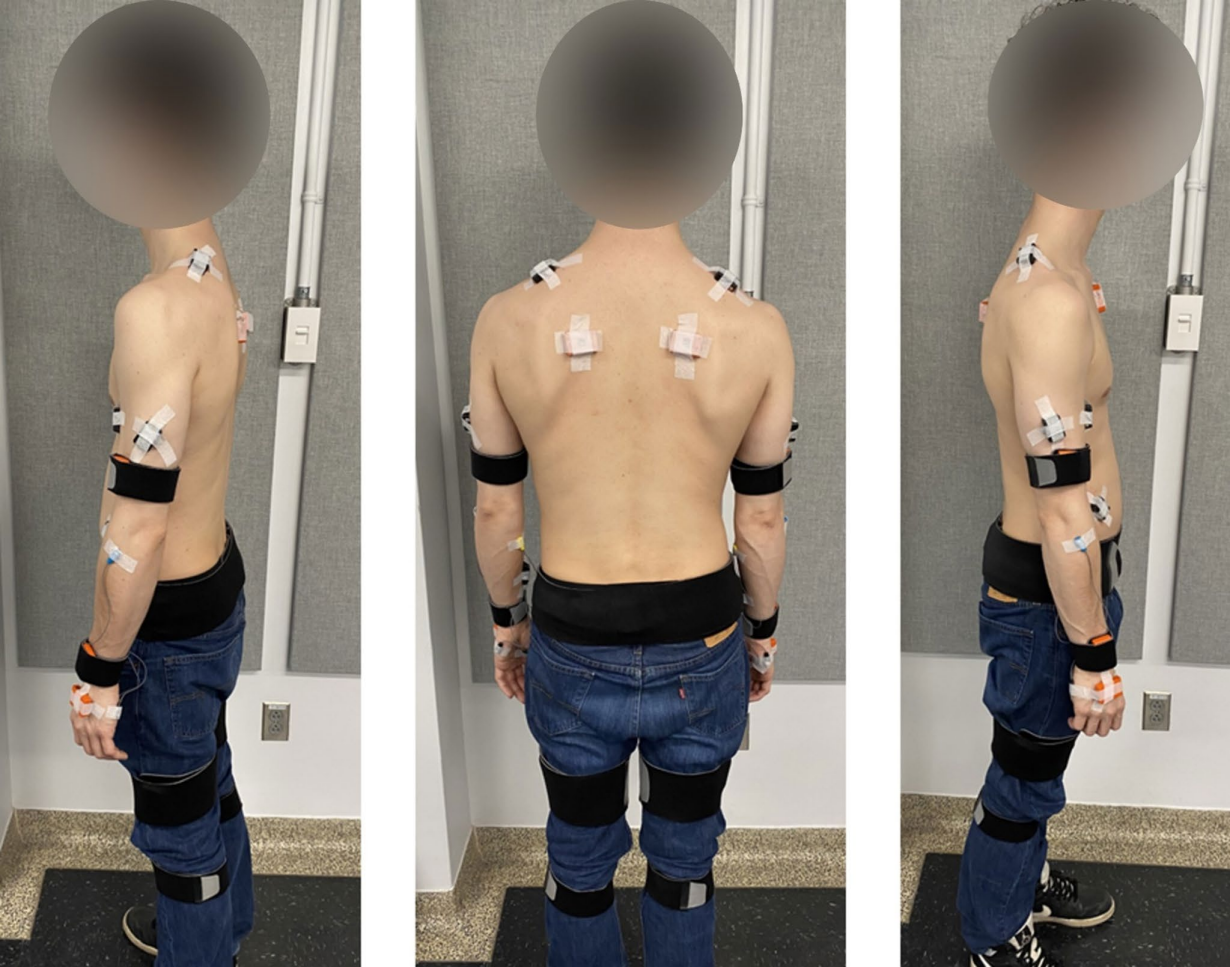
IMU placement (orange sensors secured by black Velcro straps) on the participants. Surface electromyography sensors are also visible, but the data is discussed in a complementary article (Mailly et al., 2024b).

A system composed of 17 inertial measurement units (IMUs) (XSENS, Enschede, Netherlands) recorded whole-body kinematics of participants at a sampling rate of 60 Hz. This is an accurate, non-intrusive measurement tool to capture and analyze the kinematic data addressed in this study (pianists’ ROM, posture, and distal joint angular jerk) (Adesida et al., 2019; Blair et al., 2018). Sensors were placed on the head, trunk, scapulae, upper-arms, forearms, hands, pelvis, thighs, shanks, and feet according to manufacturer recommendations (Figure 1). The hand sensors were taped onto the back of the hands using Hypafix tape to minimize interference with playing. Participants performed on a Disklavier DC7X Yamaha grand piano that recorded MIDI data (e.g., keystroke onset), which were used at a later stage for data segmentation and time-normalization purposes (MIDI data was not used for musical analysis as this was not an objective of the study). To synchronize MIDI and IMU signals for data processing, a TTL signal was automatically sent from the XSENS MVN software to sound recording software (Reaper) at the start of each recording. Using the MIDI data, the first point of analysis of IMU data was identified at 0.5 s before the onset of the first note and the last point of analysis was identified at 0.5 s after the onset of the last note for each musical excerpt (see Supplementary material for the exact start and end notes for each musical excerpt).

### 2.4 Data processing

Joint angles of the pelvis (3 degrees of freedom [DoFs]), thorax (3 DoFs), neck (3 DoFs), scapulae (3 DoFs), shoulders (3 DoFs), elbows (2 DoFs), and wrists (2 DoFs) were computed from the quaternion data of upper-limb segments using a ‘yxz’ Euler angle sequence. The pelvis and thorax joint angles were computed using quaternion data of the spine segments T8/T12 and L3/L5, respectively. There were signal artifacts with the elbow joint angles for some trials of P2 (likely from a drift in the data captured by IMUs). We therefore extracted the elbow joint angles computed by the XSENS MVN software for all trials of both participants, as the computed angles by the manufacturer’s software were absent of this artifact. This ensured consistent procedures for comparing elbow joint angles between conditions.

Based on the objectives of the study, and in line with previous research (Thompson and Luck, 2012), we computed joint angle vector norms for each of the 11 joints to obtain a global understanding of each upper-body joint angle. To calculate ROM, the minimum joint angle was subtracted from the maximum joint angle for each respective joint angle vector norm, with the top and bottom 5% outliers removed. The ROM of all joints were calculated for all musical excerpts and conditions. Each ROM value was averaged between the two trials, A and B, to give a wholistic representation for each condition. Relative difference scores to identify trends between experimental conditions were computed as follows: (Normal condition – Control condition)/Normal condition, with a positive output corresponding to a greater value in the normal condition and a negative output corresponding to a greater value in the control condition.

To analyze the effect of expressive intentions on non-neutral static posture, we computed two related variables. First, we identified periods of non-neutral posture. Cutoff angles for selected joints were identified to determine how much time pianists were engaged in non-neutral postures. These angles were: ±5° for wrist flexion/extension and ab/adduction (Oikawa et al., 2011), 5° scapular abduction (hereafter referred to as scapular elevation), 10° neck flexion (Norasi et al., 2022), and 20° thorax flexion (Claeys et al., 2016). These ranges of non-zero cutoff postures were selected given that joint postures exhibit variability during piano performance (Turner et al., 2023). The total number of time points where the joint angles exceeded these values were extracted and expressed as a percentage of the total excerpt time. Second, we addressed the static or dynamic character of non-neutral posture periods by computing the median of the angular velocity (expressed as absolute values) during the non-neutral posture periods. The angular velocity was calculated by taking the derivative of the joint rotation matrices determined from segment orientations for each joint. Velocity data were then filtered using a 2nd order zero lag Butterworth low-pass filter (Furuya et al., 2010) with a 10 Hz cut-off frequency (Monaco et al., 2017). Both the time percentage and median velocity values during non-neutral postures per joint were averaged between trials A and B and then averaged across all excerpts. To identify differences between conditions, the control condition value was subtracted from the normal condition value for both time (%) and velocity (°/s).

As the wrist was the most distal joint in our study, left/right wrist jerk was computed to address distal angular jerk. Angular acceleration and jerk were calculated by taking the time-based derivatives of the filtered angular velocity. Similar to ROM, we computed angular jerk vector norms for both wrist joints. Previous research has demonstrated that jerk-based metrics should be dimensionless (Hogan and Sternad, 2009). While a wide variety of dimensionless jerk metrics exist, and since the speed at which pianists move is an important factor during performance, we used the following equation from Hogan and Sternad (2009):


$$Jerk_{\text{dim}}=\frac{D^{3}\int_{t1}^{t2}\dddot{\theta }{\left(t\right)}^{2}dt}{{V_{\mathrm{mean}}}^{2}}$$

Where, t_1_ to t_2_ is the time interval, D is the duration, (t_2_ – t_1_), $\dddot{\theta }{\left(t\right)}^{2}$ is the squared angular jerk vector norm, V_mean_^2^ is the mean angular velocity squared, and Jerk_Dim_ is the dimensionless angular jerk. Once the dimensionless angular jerk was calculated, we computed the mean across all time points to obtain one value for each participant, musical excerpt, condition, and trial. Subsequently, trials A and B were averaged. Relative difference scores, as previously described for ROM, were also used to identify trends between experimental conditions for wrist dimensionless jerk.

To identify meaningful differences between the normal and control conditions, we computed reference thresholds for ROM, time spent in non-neutral postures, and wrist dimensionless jerk. First, the difference between trials A and B (A – B) were calculated for each participant, excerpt, and condition. We then took the mean of the absolute value of these differences across all participants, excerpts, conditions, and joints to obtain a single reference threshold value for each parameter. The reference thresholds were ±6% for ROM, ±4% for non-neutral posture, and ±19% for wrist dimensionless jerk.

## 3 Results

**Figure 2:**
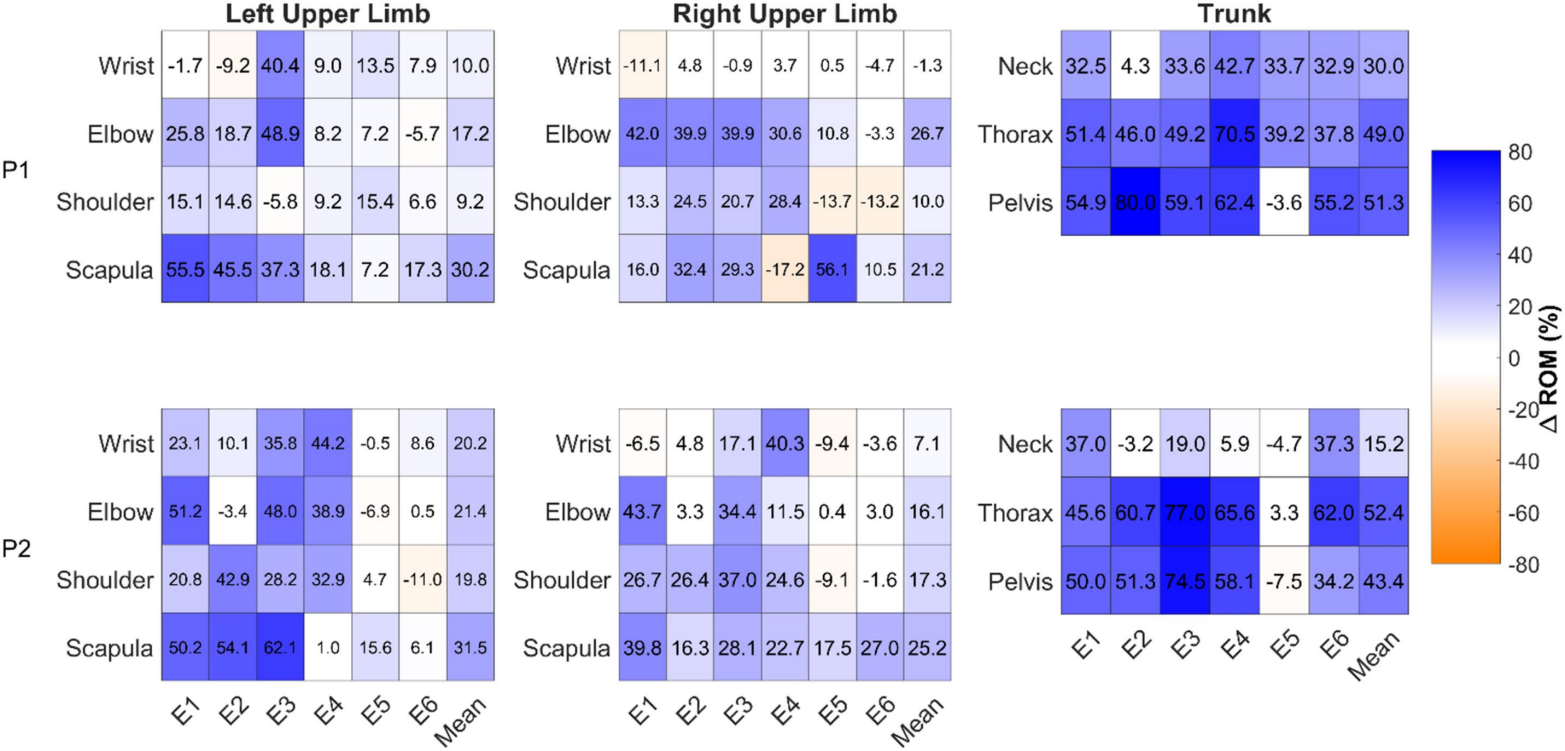
Range of motion relative differences (%) between conditions in each excerpt (E1–E6) and the mean for all 11 joints. Values were calculated using the formula (Normal – Control)/Normal. Blue represents greater ROM playing in the normal condition (positive values) while orange indicates greater ROM in the control condition (negative values). Results within the ±6% reference threshold, indicating no relevant difference between conditions (see Data Processing section), have a white background.

ROM was overall greater for P1 and P2 in the normal condition (Figure 2). This was the case both proximally and distally, with some exceptions. For instance, the right wrist exhibited different trends between excerpts and generally exhibited small differences between conditions. In E5, neck and thorax ROM were greater in the normal condition for P1 (33.7 and 39.2%, respectively) while there were no differences for P2 (−4.7 and 3.3%, respectively). For both participants, pelvis ROM during E5 exhibited no differences between conditions (P1: –3.6%; P2: −7.5%).

**Figure 3:**
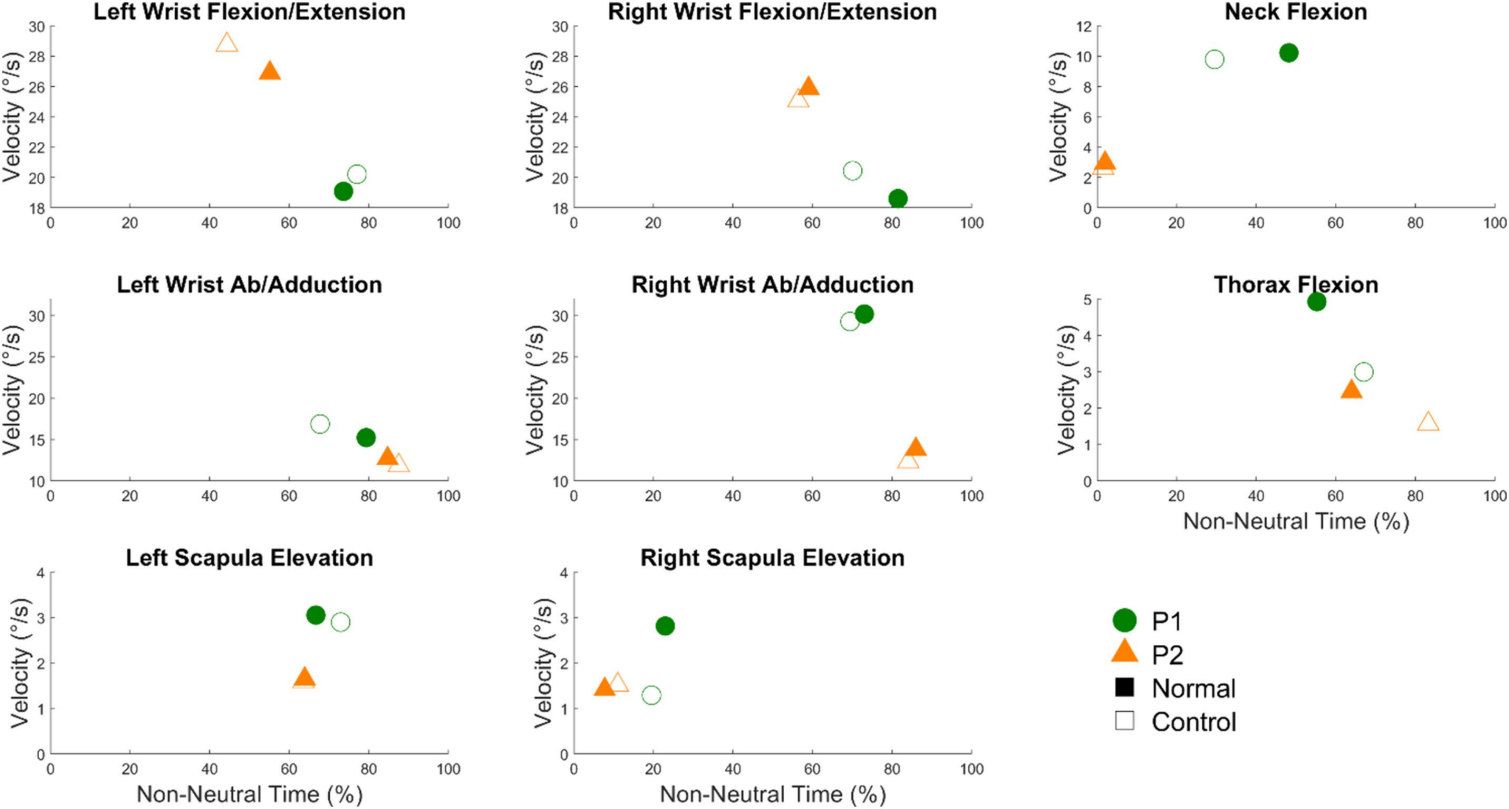
Time spent in non-neutral postures (%) on the x-axis and the median angular velocities corresponding to the times spent in non-neutral postures (°/s) on the y-axis for all 8 joints studied. Green circles represent P1 while orange triangles represent P2. Filled markers represent the normal condition and unfilled markers represent the control condition. Filled markers positioned to the right and below unfilled markers indicate more non-neutral and static postures in the normal condition, respectively. Conversely, filled markers positioned to the left and above the unfilled markers indicate more neutral and dynamic postures in the normal condition, respectively.

Figure 3 shows the time spent in non-neutral postures (%) and the median angular velocities corresponding to the times spent in non-neutral postures for both conditions. Table 1 shows the difference scores (Normal–Control). For the joints exhibiting differences between conditions, most spent more time in non-neutral postures in the normal condition. Wrist flexion/extension exhibited more non-neutral, static postures in the normal condition, specifically the right wrist for P1 (11.4%) and the left wrist for P2 (10.82%) (Table 1). For both participants, thorax flexion postures were more neutral and dynamic in the normal compared to the control condition. P1’s neck was in a more flexed, non-neutral posture in the normal condition while for P2 there was no difference between conditions because the neck spent no time in non-neutral postures (Figure 3).

**Table 1:** The difference scores between conditions (Normal—Control) for times spent in non-neutral postures (%) and the corresponding median absolute velocities (°/s).

		Time (%)	Velocity (°/s)
Left Wrist Flexion/Extension	P1	−3.40	−1.13
	P2	**10.82**	**−1.86**
Right Wrist Flexion/Extension	P1	**11.40**	**−1.84**
	P2	2.59	0.83
Left Wrist Ab/Adduction	P1	**11.59**	**−1.64**
	P2	−2.80	0.87
Right Wrist Ab/Adduction	P1	3.60	0.91
	P2	1.90	1.52
Left Scapula Elevation	P1	**−6.22**	**0.15**
	P2	0.31	0.06
Right Scapula Elevation	P1	3.45	1.52
	P2	−3.28	−0.10
Neck Flexion	P1	**18.70**	0.43
	P2	0.47	0.35
Thorax Flexion	P1	**−11.78**	**1.93**
	P2	**−19.29**	**0.89**

Relative to the normal condition, positive values for time and velocity represent non-neutral and dynamic postures, respectively. Time difference results exceeding the ±4% reference threshold, indicating relevant differences between conditions, are bolded.

**Figure 4:**
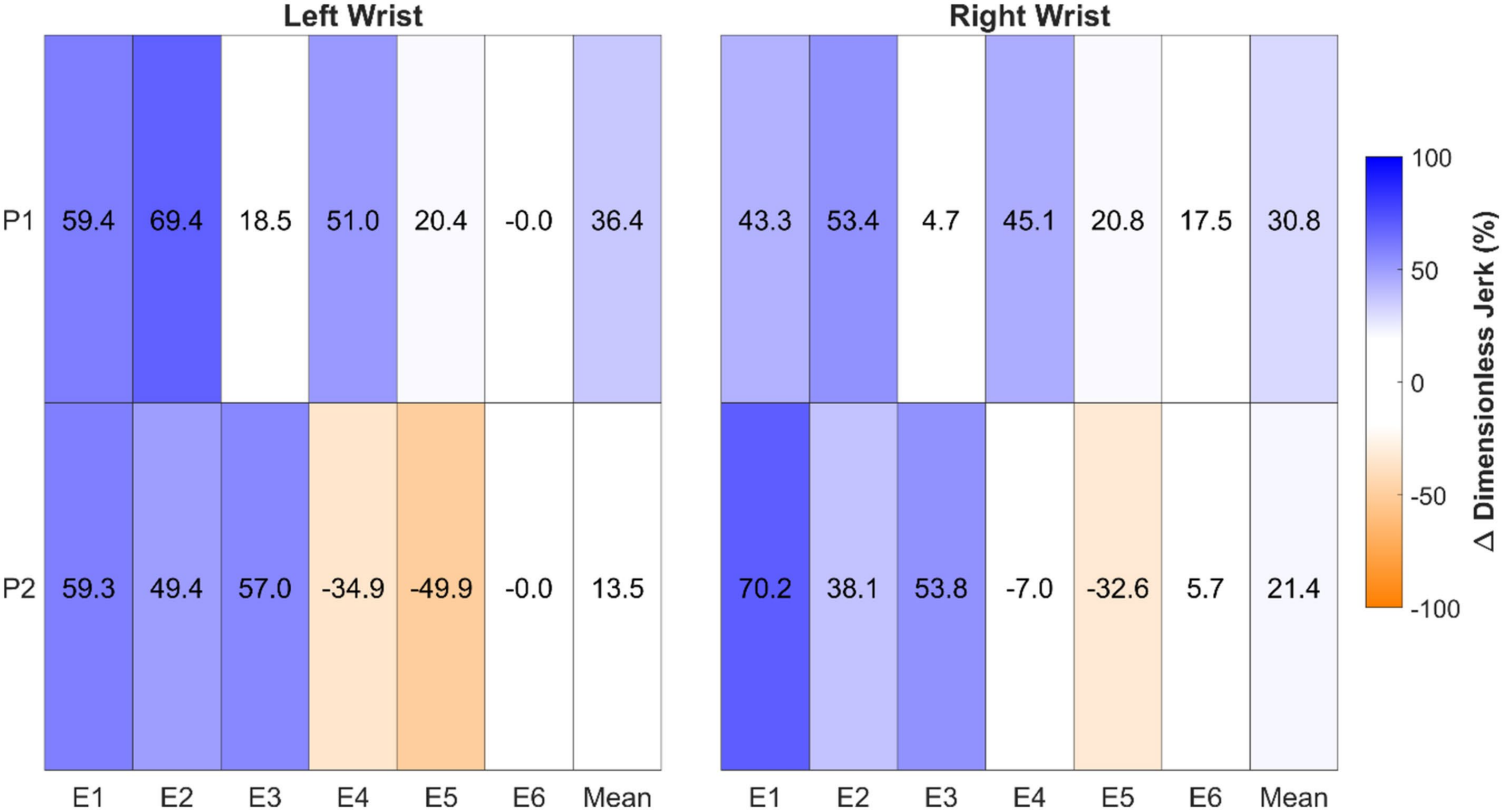
Dimensionless jerk relative differences (%) between conditions in each excerpt (E1-E6) and the mean for the left and right wrists. Values were calculated using the formula: (Normal—Control)/Normal. Blue represents greater jerk in the normal condition (positive values) while orange represents greater jerk in the control condition (negative values). Note: Lower jerk indicates smoother movements. Results within the ±19% reference threshold, indicating no relevant difference between conditions (see Data Processing section), have a white background.

Wrist jerk was overall greater in the normal condition (Figure 4), however, this changed based on the type of musical excerpt. For lyrical excerpts (E1–E4), both participants’ left and right wrist jerk was greater in the normal condition, with the exception of E3 and E4 for P1 and P2, respectively. In terms of virtuosic excerpts (E5–E6), both participants exhibited no relevant differences between conditions for both wrists, with the exception of E5 for P2 (left: −49.9%; right: −32.6%). Notably, there were no differences between conditions in E6 for left wrist jerk for both participants (0%).

## 4 Discussion

The current exploratory case study is the first to document how expressive intentions can influence joint angle kinematics in the entire upper-body kinematic chain of expert pianists. The objective was to analyze the influence of pianists’ expressive intentions across different musical contexts on both proximal and distal joint range of motion (ROM), postures of joints that are commonly injured in pianists, and wrist angular jerk. Overall, both proximal and distal ROM increased for both participants when incorporating expressive intentions, however, changes were greater in proximal joints. In terms of posture, when incorporating expressive intentions, participants exhibited more static, non-neutral wrist postures (right and left wrist for P1 and P2, respectively) while the thorax exhibited more dynamic, neutral postures. Incorporating expressive intentions, generally, also led to jerkier wrist movements in lyrical excerpts (E1–E4), however, for more virtuosic excerpts (E5–E6), there were less relevant differences and, when present, varied between participants. In a musical context where the left hand played loud, fast, staccato notes (E6), there were 0% differences in left wrist jerk between conditions.

### 4.1 Range of motion

Our study quantified joint angle kinematics to assess how expressive intentions influence movement strategies rather than quantifying the distance markers move when placed on segments. Both proximal (head, trunk, scapulae, shoulders) and distal (left wrist and both elbows) ROM were greater for both participants when performing with expressive intentions. Our results agree with previous research that assessed linear motion of markers placed on joints, which has suggested that performing with greater expressivity leads to more motion for the head (Castellano et al., 2008; Massie-Laberge et al., 2019; Thompson and Luck, 2012), trunk (Nakahara et al., 2010), and shoulder girdle (Thompson and Luck, 2012). For the wrists and elbows, our results partially agree with the literature. Thompson and Luck (2012) observed that markers placed on the elbows and wrists moved more in experimental conditions when pianists performed with greater expressivity (i.e., exaggerated) when analyzing each individual measure of a piece of music. Differences between conditions were less pronounced for distal segments compared to proximal segments likely due to their sound production role (and task-constrained fingertip position), suggesting that expressive intentions may have less impact on distal segments (Thompson and Luck, 2012). The results of the current study suggest that, for these two pianists, expressive intentions influence distal and proximal joints equally, as differences between conditions for wrist and elbow ROM were sometimes greater than ROM for proximal joints, depending on the musical excerpt. Different phenomena could explain this influence on distal joints. Firstly, greater distal ROM might result from changes in the proximal kinematic chain. It has been shown in piano performance that trunk motion can contribute to creating distal velocities (Verdugo et al., 2020) and initiate distal movements via a proximal-to-distal movement sequencing (Verdugo et al., 2020; Verdugo et al., 2022a). Additionally, changing elbow movements can influence finger kinematics due to a change in forearm orientation relative to the keyboard (Furuya et al., 2011). In fact, changes in the proximal kinematic chain of pianists have been shown to increase distal kinematic variability (Turner et al., 2023). Therefore, changes in distal movement strategies might arise from changes in the proximal kinematic chain that are directly a result of expressive intentions. Secondly, these changes in distal joint ROM might also result from expressive intentions directly impacting distal joints themselves. In other words, it is possible that greater ROM for the left wrist and both elbows might carry ancillary gestural functions directly resulting from expressive intentions in addition to a sound production function. Notably, incorporating expressive intentions did not always result in greater right wrist ROM, with little to no increases for P1 across all excerpts. For P2, this was also the case except for E3 and E4. These differences between participants for E3 and E4 suggest that in certain musical contexts, individualized playing techniques or playing approach might impact how expressive intentions influence right wrist joint movement. While the right hand is often more responsible for musical phrasing of the melody, which requires different temporal and dynamic (volume) characteristics between the hands (Kim et al., 2021), it could be that certain, individualized movement strategies for “voicing” a melody are more influenced by pianists’ expressive intentions.

In a complementary analysis involving the same two participants and conditions where muscle activity was evaluated, P1 exhibited greater proximal and distal muscle activity when integrating expressive intentions, while for P2, no clear trends were observed across trials and conditions (Mailly et al., 2024b). These findings suggest that greater ROM due to expressive intentions (as shown by both participants in the current study) may not necessarily equate to greater muscle activity. Consequently, higher muscle activations of P1 found by Mailly et al. (2024b) might not be directly attributed to the reported increase in ROM but to frequent occurrences of isometric contractions, as theorized by Verdugo, Ceglia, Frisson et al. (2022b). Such differences between kinematic and physiological embodiment of expressive intentions might have implications for how each pianist could be exposed to risk factors of PRMDs.

For E5 (1st movement of Beethoven Sonata op. 111), the only musical context with continuous, fast parallel sixteenth-notes and no rests, neck and thorax ROM was different between participants. P2 exhibited minimal differences in neck and thorax ROM between conditions possibly due to higher spatiotemporal constraints (in other words, higher technical demands). On the contrary, P1 exhibited greater ROM in the normal condition, possibly due to interpretation of the musical score regardless of spatiotemporal constraints. This might suggest a greater ease of movement, the opposite to a “freezing” effect of joints, commonly observed when novices perform complex motor skills (Guimarães et al., 2020). These findings suggest that in musical contexts where spatiotemporal constraints are high, pianists might still use greater ROM of proximal movements in relation to their musical intentions. However, in the musical context of E5, the greater proximal ROM might not apply to the pelvis as it showed no differences between conditions for both participants. Considering all of the ROM results, pianists’ gestures, whether proximal or distal, might serve different functions that are tied to musical demands. However, this might not be necessarily generalizable.

### 4.2 Posture

In the current study, posture was influenced by expressive intentions. Differences between participants were observed for wrist flexion/extension, where P1’s right wrist, and P2’s left wrist postures, were more non-neutral and static when performing with expressive intentions (P1: 11.40%; P2: 10.82%). Some studies suggest that pianists should maintain neutral wrists to reduce risk of injury (Oikawa et al., 2011). Additionally, ergonomic recommendations such as the Rapid Upper Limb Assessment (RULA) (McAtamney and Nigel Corlett, 1993) suggest neutral postures can help reduce risk of musculoskeletal disorders during repetitive movements. These results suggest that incorporating expressive intentions might contribute to static, non-neutral wrist postures that are associated with increased risk of PRMDs. However, this might not necessarily impact both wrists and may actually be participant dependent. Similarly, neck postures were different between participants as P1’s neck was in a more flexed, non-neutral posture in both conditions while P2’s neck was in a neutral posture in both conditions. These differences between participants might be because postures are highly idiosyncratic for all musicians (Shoebridge et al., 2017) and might be dependent on methods of piano pedagogy and approaches to piano technique.

The notable similarity between participants was that thorax flexion postures were more dynamic and neutral for both participants when playing with expressive intentions. Interestingly, Wong et al. (2022) found that when playing a musical excerpt, trunk angles were more flexed (i.e., non-neutral) in an “exaggerated,” ‘more expressive’ condition compared to a “deadpan,” ‘less expressive’ condition. Our results are in opposition to these results. The experimental protocol used in the present study did not include exaggerated or deadpan conditions. Participants played the selected musical excerpts in a normal condition (integrating their expressive intentions) and in a control condition where they performed the excerpts by focusing only on the musical features written by the composer (which usually include manipulation of specific musical parameters such as sound intensity). We believe the two conditions used in our study are more representative of actual practice situations, where pianists may practice both their personal interpretation of the score and a more objective version without expressive intentions during the learning/practice process before a performance. Therefore, unlike the results found by Wong et al. (2022), our results suggest that more objective practice increases the time spent in a flexed and static trunk posture. In addition, our study included a greater variety of musical contexts, which possibly explains discrepancies between our results and previous findings. Considering wrist, neck, and thorax postural results, expressive intentions may impact proximal and distal postures differently. This presents a new perspective and avenue in which researchers, piano teachers, and health practitioners can address posture in relation to music expression in piano performance. Creating connections between pianists’ expressive intentions and movement-based injury prevention strategies might make concepts of posture and biomechanics practically meaningful and help promote deeper levels of understanding in pianist communities.

### 4.3 Movement jerk

We evaluated how expressive intentions impacted wrist jerk, which is a kinematic parameter related to risk of injury. As both participants, on average, exhibited greater wrist jerk when incorporating expressive intentions, this generally suggests that expressive intentions could contribute towards greater risk of PRMDs in the wrists. Compared to more proximal joints, the wrists are functionally more susceptible to higher levels of jerk due to less biomechanical impedance (Salmond et al., 2017). As wrist jerk generally increased for lyrical, less virtuosic excerpts (E1-E4), this functional characteristic might allow wrist jerk to be more influenced by expressive intentions in musical contexts where spatiotemporal constraints are not high. On the contrary, smaller changes or no changes in wrist jerk for virtuosic musical excerpts (E5–E6) might be due to higher spatiotemporal constraints. This suggests that incorporating expressive intentions in musical contexts that require more technical skill (i.e., more virtuosity) might not have a relevant impact on wrist jerk. However, these trends between virtuosic and non-virtuosic excerpts were not always consistent between participants (especially for E3, E4, and E5) suggesting that individualized influences in wrist jerk might still occur possibly because of playing approach and/or the intended musical outcome. Remarkably, both participants exhibited 0% differences between conditions in left wrist jerk for E6, which required that the left hand played a series of loud, rapid notes in a detached manner (sixteenth notes in a *staccato* articulation). Given that playing fast and with *staccato* articulations are musical contexts thought to increase exposure to risk factors of PRMDs (Mailly et al., 2024a), considering expressive intentions in relation to wrist jerk might not mitigate nor increase pianists’ exposure to PRMDs when playing fast and *staccato*. Wrist jerk could be an interesting parameter to study further in terms of repetitive movements in this musical context. These overall findings potentially offer insights on how pianists’ expressive intentions, in combination with certain musical excerpts/contexts, might elicit changes in movement smoothness and possibly increase exposure to risks of PRMDs.

### 4.4 Limitations and future research

There are some limitations in the current case study. Results cannot be generalized to a larger population due to the sample size of two participants. Analyzing how two pianists’ ROM, posture, and angular jerk changed when incorporating expressive intentions provided individual insights that future work can use as a framework. Recruiting more participants is the next step to continue building our understanding of the relationship between pianists’ expressive intentions and exposure to risk factors of injury. Additionally, the data collection occurred in a controlled environment and not in a live performance setting. Comparing the experimental conditions related to expressive intentions in a live performance and involving more pianists could be an interesting avenue for further exploration into how expressive intentions impact pianists’ proximal and distal movement strategies. Based on our findings, we would expect that not only the choice of repertoire period but also of the specific musical context itself could lead to different results. While this study was the first to investigate the link between expressive intentions and exposure to kinematic risk factors of injury, such as poor posture and jerky movements, more research is necessary on not only these parameters but also on movement dynamics (i.e., joint torques), which are also related to risk of PRMDs.

## 5 Conclusion

This exploratory case study is the first to suggest a connection between how expressive intentions can influence pianists’ movement strategies both in terms of performance and exposure to risk factors of injury. Both proximal and distal joint kinematics of the two pianists in the study exhibited increased ROM when incorporating expressive intentions, showing that expressive intentions can affect the whole kinematic chain and not just proximal joints/segments. When incorporating expressive intentions, participants exhibited more static, non-neutral wrist postures (right and left wrist for P1 and P2, respectively) suggesting an increased risk of distal injury. In contrast, and contrary to previous studies, the thorax exhibited more dynamic, neutral postures, suggesting a reduced risk of proximal injury. Expressive intentions may, therefore, impact proximal and distal postures differently. Incorporating expressive intentions also led to jerkier, less smooth wrist movements in lyrical musical excerpts, however, in more virtuosic excerpts, there were generally no differences between conditions. These results provide an understanding of how expressive intentions impact the entire kinematic chain, highlighting the implications of the subjective features of music expression in relation to exposure to risk factors of PRMDs.

## Data Availability

The raw data supporting the conclusions of this article will be made available on request.
