# Supplementary material for: Impact of expressive intentions on upper-body kinematics in two expert pianists
Source: Front Psychol. 2025 Jan 13;15:1504456. doi: 10.3389/fpsyg.2024.1504456 (PMC11770054; doi:10.3389/fpsyg.2024.1504456)
Supplement: Supplementary file 1 [file Data_Sheet_1.docx]

Supplementary Material

The beginning and/or end of the sections played for the 6 musical excerpts (E1-E6) are marked by red brackets.


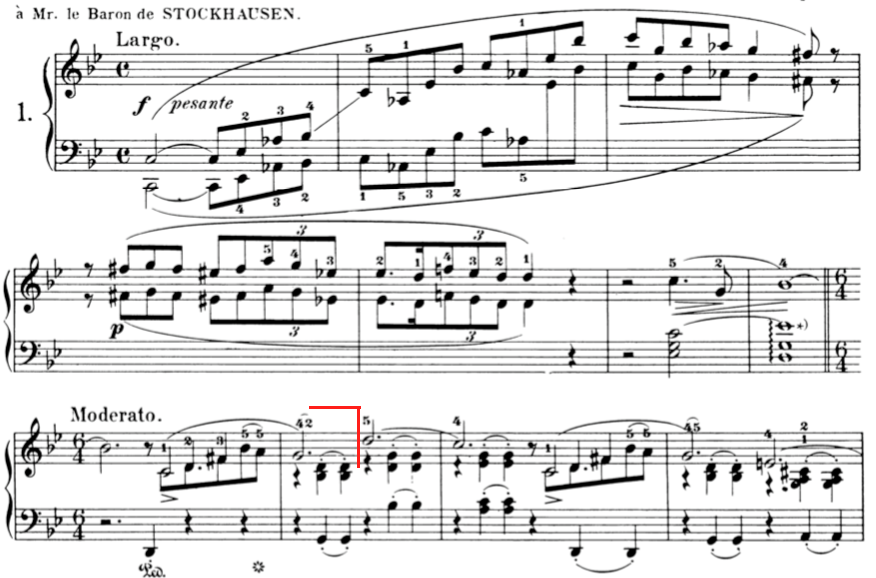


**Supplementary Figure 1.** E1: Ballade Op. 23 No. 1 in G minor [measures: 1-9] by F. Chopin.


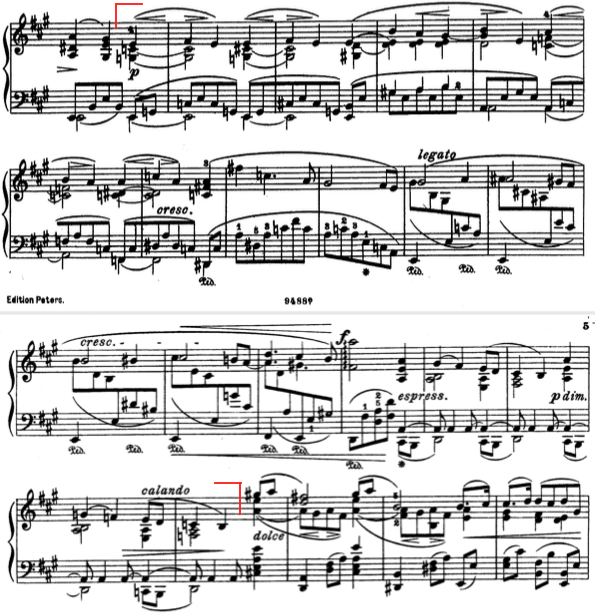


**Supplementary Figure 2.** E2: Intermezzo Op. 118 No. 2 in A major [measures: 17-34] by J. Brahms.


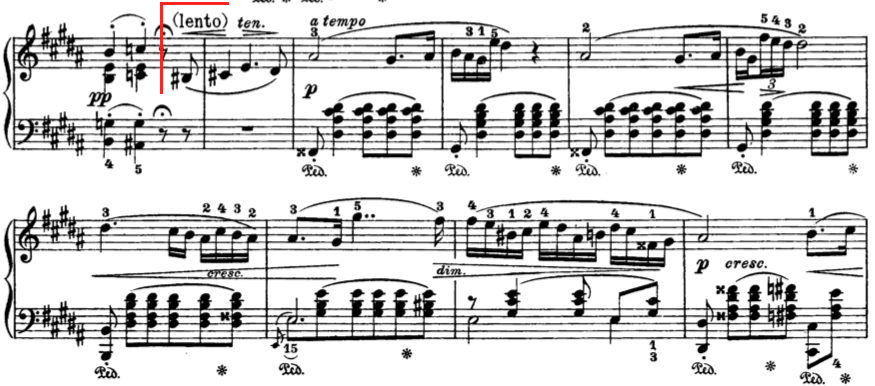


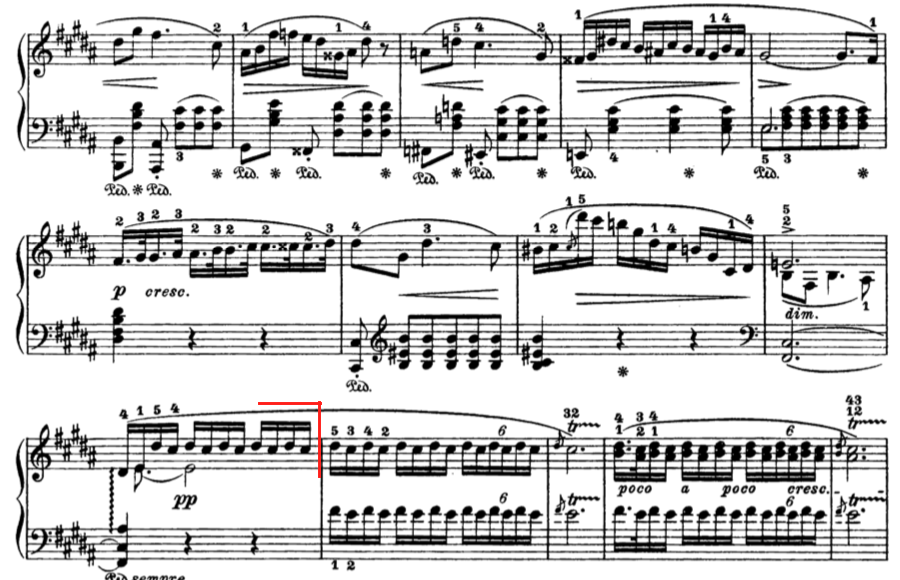


**Supplementary Figure 3.** E3: Polonaise-Fantasy Op. 61 in A-flat major [measures: 181-199] by F. Chopin.


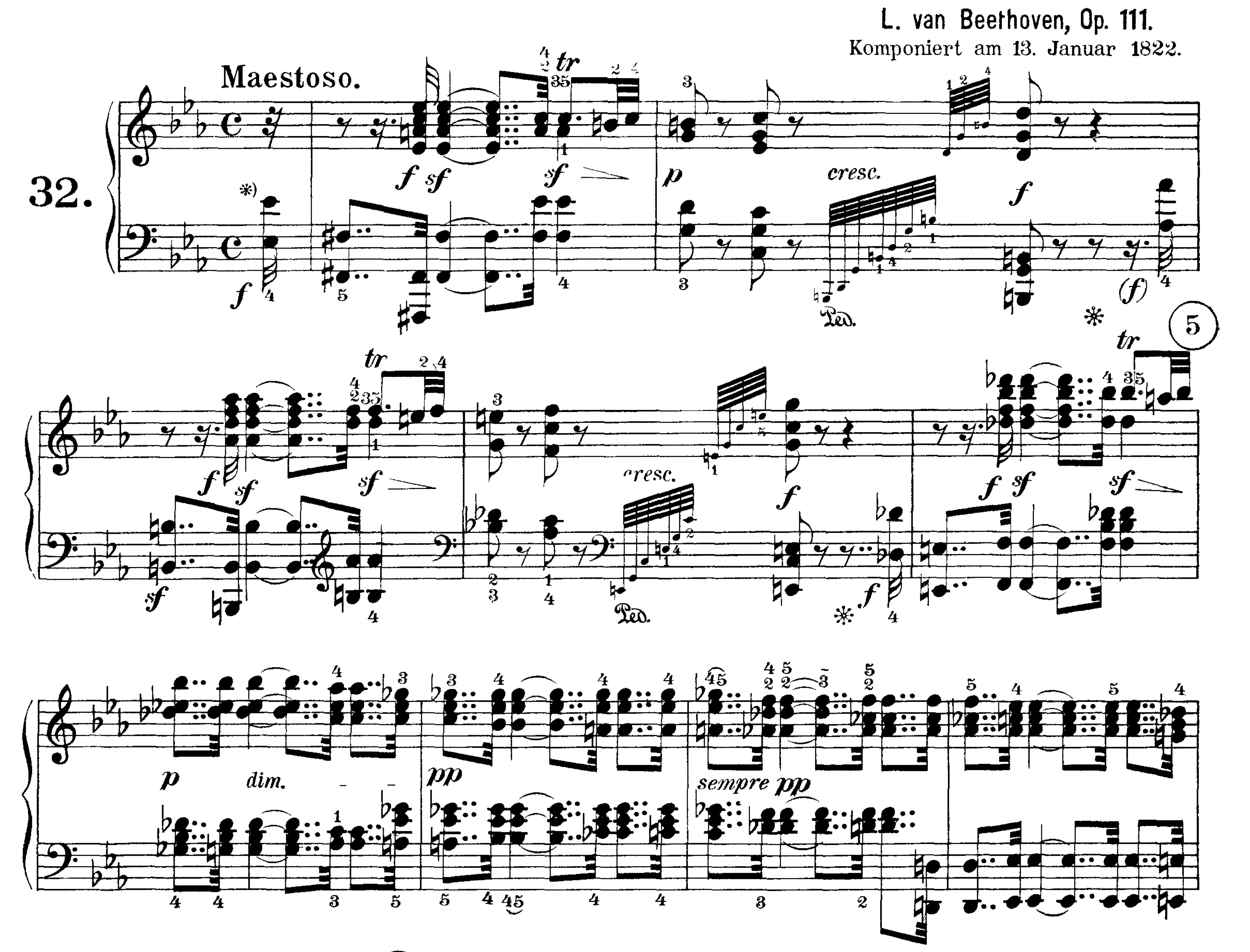


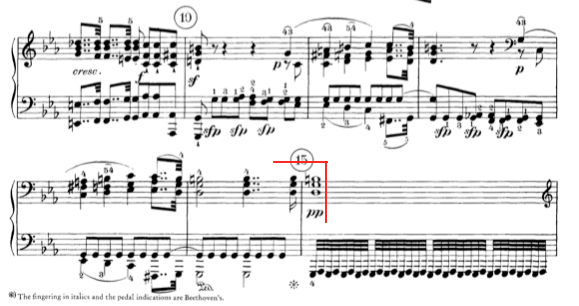


**Supplementary Figure 4.** E4: Sonata Op. 111 in C minor, 1^st^ movement [measures: 1-16] by L. v. Beethoven.


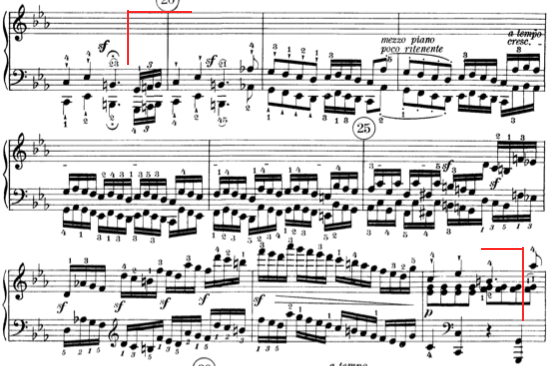


**Supplementary Figure 5.** E5: Sonata Op. 111 in C Minor, 1^st^ movement [measures: 21-29] by L. v. Beethoven.


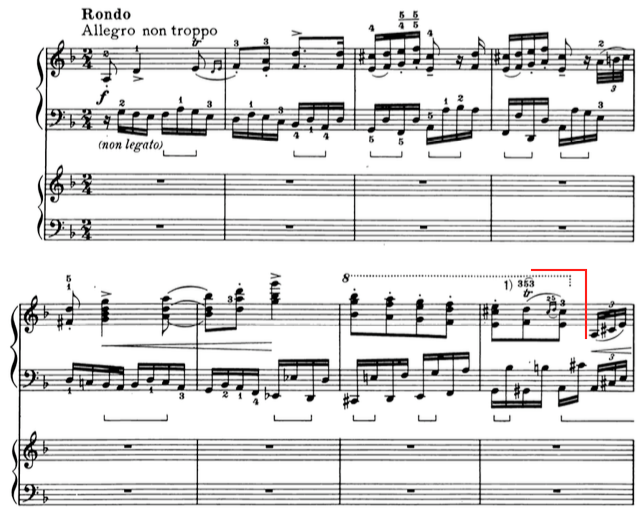


**Supplementary Figure 6.** E6: Concerto for Piano Op. 15 No.1 in D minor, 3^rd^ movement [measures: 1-8] by J. Brahms.
